# State of the Art Bowel Management for Pediatric Colorectal Problems: Anorectal Malformations

**DOI:** 10.3390/children10050846

**Published:** 2023-05-08

**Authors:** Elizaveta Bokova, Wendy Jo Svetanoff, Joseph J. Lopez, Marc A. Levitt, Rebecca M. Rentea

**Affiliations:** 1 Comprehensive Colorectal Center, Department of Surgery, Children’s Mercy Hospital, Kansas City, MO 64108, USA; eobokova@gmail.com (E.B.);; 2 Division of Colorectal and Pelvic Reconstruction, Children’s National Medical Center, Washington, DC 20001, USA; 3 Department of Surgery, University of Missouri-Kansas City, Kansas City, MO 64108, USA

**Keywords:** bowel management, anorectal malformation, ARM, imperforate anus, antegrade enema, Malone, laxative, enema, constipation, incontinence

## Abstract

Up to 79% of patients with anorectal malformations (ARMs) experience constipation and/or soiling after a primary posterior sagittal anoplasty (PSARP) and are referred to a bowel management program. We aim to report the recent updates in evaluating and managing these patients as part of the manuscript series on the current bowel management protocols for patients with colorectal diseases (ARMs, Hirschsprung disease, functional constipation, and spinal anomalies). The unique anatomic features of ARM patients, such as maldeveloped sphincter complex, impaired anal sensation, and associated spine and sacrum anomalies, indicate their bowel management plan. The evaluation includes an examination under anesthesia and a contrast study to exclude anatomic causes of poor bowel function. The potential for bowel control is discussed with the families based on the ARM index calculated from the quality of the spine and sacrum. The bowel management options include laxatives, rectal enemas, transanal irrigations, and antegrade continence enemas. In ARM patients, stool softeners should be avoided as they can worsen soiling.

## 1. Introduction

Anorectal malformations (ARMs) occur due to abnormal hindgut formation in 1 in every 4000–5000 newborns, with a slightly higher incidence in males [1,2,3,4,5]. The disease occurs due to cloaca and urorectum dysmorphogenesis in early fetal life [6]. More than 75% of ARM patients have an associated anomaly [3], including VACTERL association (vertebral, anorectal, cardiac, tracheoesophageal, renal, and limb anomalies) present in 70% of patients with an ARM [7,8,9,10,11].

Results of surgical and medical interventions require a long-term follow-up as the functional results are unknown in early childhood. Patients with an ARM are likely to have several medical or surgical interventions during their lifetime. Additional surgeries may be needed as children grow, requiring additional maintenance care and multiple surgical specialists. The average child with an ARM has 32 inpatient and 126 outpatient healthcare days in the first 5 years of life [12]. Up to 79% of ARM patients have constipation after a primary posterior sagittal anorectoplasty (PSARP) [13], up to 48% experience soiling [14], and require bowel management to achieve continence with 1–3 bowel movements per day and 1 or fewer accidents per week, according to the Rome IV criteria (Table 1) [15,16,17,18,19].

The understanding of the potential for bowel control and the protocols for diagnosis and management of these children have changed over the last years with a constantly increasing amount of research data published on the topic [20]. Our goal is to review the current practice in bowel management for this group of patients. The current article is part of a manuscript series on updates in bowel management in different groups of colorectal patients (ARM, Hirschsprung disease, spinal anomalies, and functional constipation).

## 2. Methods

A state-of-the-art review of literature published before March 2023, in Medline/PubMed, Google Scholar, Cochrane, and EMBASE databases, including original studies, meta-analyses, randomized controlled trials, and systematic reviews, focuses on manuscripts and books published over the last 5–10 years in English. Relevant keywords and MeSH terms were used to ensure broad coverage of the topic. Search keywords included: “anorectal malformation”, “bowel management”, “imperforate anus”, “constipation”, “fecal incontinence”, “potential for continence”, “enema”, “laxatives”, and “irrigation”. The reference lists of the retrieved articles were checked for other relevant articles not found during the initial search. Articles providing novel insights or addressing current challenges in the field were prioritized. Ninety-three of the selected articles were included in the current review. The data were reported in a narrative format focusing on the recent updates on the bowel management of patients with ARMs to provide an in-depth, stepwise protocol for bowel management that considers the patient’s potential for bowel control and serves as a valuable resource for physicians treating these children.

## 3. Anatomic Considerations

Anorectal malformations are complex congenital anomalies that require an individualized strategy of care for each step of the treatment process. These steps, which include preoperative evaluation, surgical reconstruction, and postoperative care, are unique and vitally important to overall patient outcomes.

### 3.1. Initial Repair

To prevent the passage of stool to the urinary tract, patients with an ARM require an ostomy creation at birth [21]. A Turnbull-type diverting loop or a divided sigmoid colostomy with a mucous fistula diverts the stool, allowing for a distal colostogram in the future to define the anatomy prior to reconstruction [21]. Based on the colostogram, the type of malformation, according to the Krickenbeck classification system [21] and the optimal operative approach, are defined. The imaging indicates the access for the repair (PSARP vs. laparoscopic-assisted anorectoplasty), reveals the fistula if one is present [22], and protects the ultimate anorectal repair.

### 3.2. Postoperative Assessment

Following surgical repair, the assessment of a child with ongoing bowel issues requires an understanding of the original malformation, the child’s anatomic status following repair, the quality of the sacrum, and the spine, and a conclusion on their ultimate continence potential [23]. A combination of medical records and anorectal examination under anesthesia (EUA) determines the anatomy and assesses for any other urogenital anomalies [24]. 

When a child is referred for management of constipation or soiling, obtaining a comprehensive medical and surgical history is crucial, including whether the patient has had a previous colostomy and the type of prior ARM repair [24]. The next step is a workup to assess the anatomy. It includes a plain abdominal film to evaluate stool burden and an EUA to assess sphincter function, the location of the neoanus within the sphincter complex, and identify the presence of an anal stricture or rectal prolapse [24]. Cystoscopy and vaginoscopy in collaboration with a urologist and a gynecologist determine if additional anomalies exist or identify a remnant of the original fistula (ROOF) [25,26]. Imaging studies should include a pelvic and spinal MRI to reveal unidentified spinal cord anomalies or presacral masses and a contrast enema to specify the colonic anatomy, strictures, or other possible complications following the patient’s previous surgical procedures [24]. In children younger than 6 months, spinal ultrasound is used instead of MRI to screen for spinal anomalies [27].

### 3.3. Anatomic Characteristics

Normally, bowel control is regulated by four factors: (1) anal sensation—the ability to differentiate between solid, liquid, and gas; (2) function of the sphincters—the ability to squeeze and relax the sphincters at the appropriate time; (3) rectosigmoid motility—the movement of the colon, too fast or too slow; and (4) reservoir function (proprioception)—the ability to detect the stretch of the rectum, which triggers the need to squeeze the external (voluntary) anal sphincters [28]. It is important to keep in mind that in ARM patients (except those with rectal atresia and anal stenosis), the anal canal is not embryologically formed, and thus, they do not have proper anal sensation; the rectal mucosa is attached to the anal skin during the operation leading to postoperative fecal incontinence [21]. 

Children with an ARM often have the entire length of their colon preserved with modern surgical procedures. Maximal saving of the colon allows for higher water absorption, and therefore, more solid stools that are easier to detect [29]. To take advantage of proprioception, they require a timed and bulky rather than liquid stool. Rectosigmoid resection in children with an ARM worsens soiling, as after resection, the reservoir function of the rectum would be lost, and the patient would have more difficulty detecting the stretch of the neorectum. In such a case, the high-amplitude propagated contractions (HAPCs) of the sigmoid or more proximal colon [30] will reach the perineum directly, resulting in frequent uncontrolled stools.

## 4. Potential for Continence

When discussing long-term outcomes, parents seek information about the likelihood of achieving fecal continence [24]. A recent study of ARM types, not including cloaca, found that fecal incontinence occurred in 42–48% of patients with severe fecal incontinence (Krickenbeck Grade 2 and 3) present in 36–37% of patients [14,21]. Several characteristics allow us to predict the potential for bowel control for each patient.

### 4.1. Type of Malformation

A multi-institutional cohort study of children with ARMs identified that the anatomic type of ARM was the most predictive tool for future continence potential and subsequent need for a bowel management program (BMP) [31]. Patients with less severe ARM subtypes (perineal fistula, recto-bulbar fistula, recto-vestibular fistula, no fistula, and rectal stenosis) had a higher continence potential, with some subtypes approaching a 100% expectation of continence [31].

### 4.2. Sacral Development

ARMs develop because of an embryologic arrest of pelvic musculature development, commonly associated with anomalies of the sacrum and spine [13,17,32]. Poor muscular development negatively affects the patient’s ability to hold stool and leads to fecal incontinence [23]. The presence of these anomalies can affect the potential of a child to achieve bowel control [23].

The determination of the amount of sacral hypodevelopment is aided by an objectively determined sacral ratio (SR), which compares the sacrum size with the fixed bony parameters of the pelvis in both anteroposterior (AP) and lateral radiographic views [33,34]. This value is typically calculated based on radiographic studies, including sacral radiogram and distal colostogram. Alternatively, MRI scans can be utilized for the measurements with good inter-rater reliability shown in the MRI measurement of SR [35]. 

**Figure 1:**
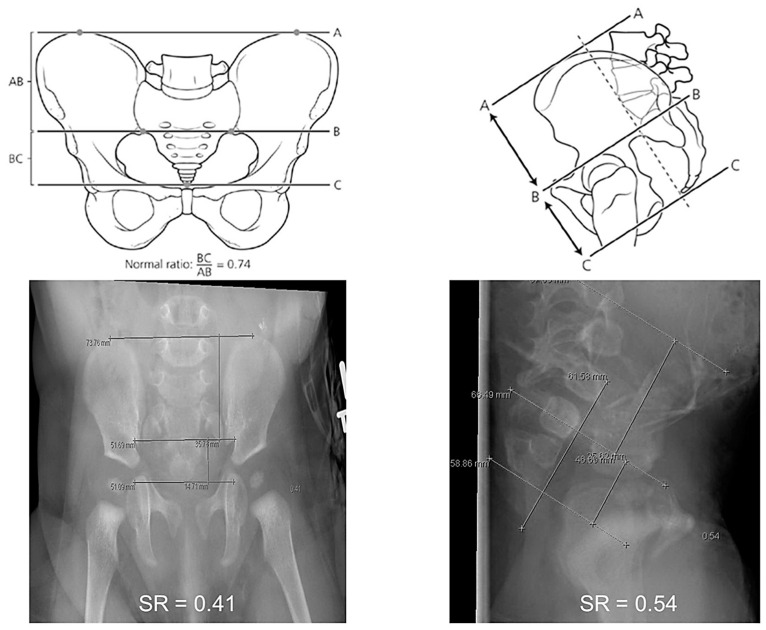
Calculation of the sacral ratio. Three horizontal lines are drawn: at the top of the iliac crests (A), at the inferior point of the sacroiliac joints (B), and at the tip of the coccyx (C). The sacral ratio is calculated by dividing the distances between BC and AB lines. Reprinted from Ref. [34], with permission from Elsevier.

The SR is calculated using three horizontal lines: (1) at the top of the iliac crests; (2) at the inferior point of the sacroiliac joints; and (3) at the tip of the coccyx. The calculation process is depicted in Figure 1 [34]. The lateral view is more reliable than the AP view, as on the latter, the sacrum can look shorter due to the pelvic tilt [33]. Based on the sacral ratio, the patients can be divided into three groups: (1) SR less than 0.4, which indicates poor potential for bowel control; (2) SR 0.4–0.69; and (3) SR 0.7 or higher, which is associated with a higher likelihood of fecal continence [23]. These measurements can assist the surgeon when counseling families on bowel management prognosis [36].

### 4.3. Spinal Anomalies

Abnormalities of the spinal cord are present in 14–57% of ARM patients and are not always associated with sacral malformations [37,38,39,40]. Some authors report an association between the severity (or “level”) of the anorectal malformation and spinal lesions [39,40,41,42,43,44]. However, this statement is discussible as a high incidence of the tethered cord was reported in patients with “low” ARMs as well [37]. 

The role of spinal anomalies on the long-term outcomes in children with an ARM remains unclear. Kyrkland et al. conducted a study that showed that patients with non-severe spinal anomalies (excluding extensive malformations and myelomeningocele) have similar functional outcomes when compared to ARM patients with no spinal anomalies [45]. Another study reported that spinal anomalies do not affect outcomes in patients with an ARM, leading to the conclusion that only the type of ARM is important for bowel control in the future [46]. Other authors consider spinal and sacral anomalies as crucial factors contributing to the potential for continence [23,46]. Di Cesare et al. showed that the presence of a tethered cord and the sacral ratio are the key characteristics affecting the functional outcomes [46]. 

### 4.4. ARM Index

**Figure 2:**
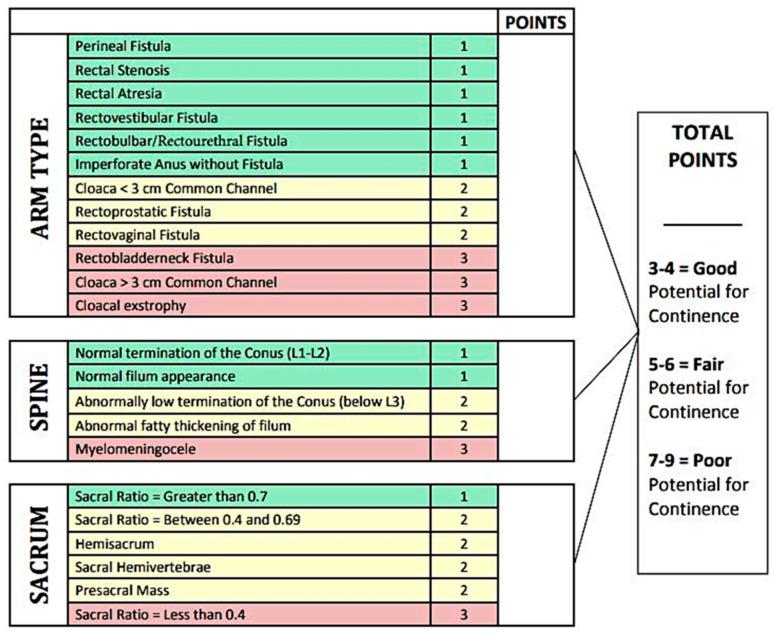
Prognosis of bowel control based on the type of anorectal malformation, spinal anatomy, and sacral development. This scale puts patients at a high, medium, or low risk for fecal continence. The more complex type of ARM or the less developed the sacrum or spinal cord, the lower the likelihood that the patient will develop fecal continence without the assistance of a bowel management regimen. ARM—anorectal malformation. Reprinted from Ref. [23], with permission from Elsevier.

Based on the data described above, both the type of ARM and spinal and sacral anomalies should be taken into consideration when counseling families about bowel management expectations. The ARM continence index has been developed to assess the patient’s potential for bowel control in continence prediction counseling based on their ARM type, quality of sacrum, and spine (Figure 2) [23,36,47]. There are three factors included: (1) the type of anorectal malformation; (2) the quality of the spine; and (3) the sacral development. This scale puts patients at a high, medium, or low risk for fecal continence. The more complex the type of ARM or the less developed the sacrum or spinal cord, the lower the likelihood that the patient will develop fecal continence and will not need the assistance of a bowel management regimen [23].

## 5. Bowel Management Options

**Figure 3:**
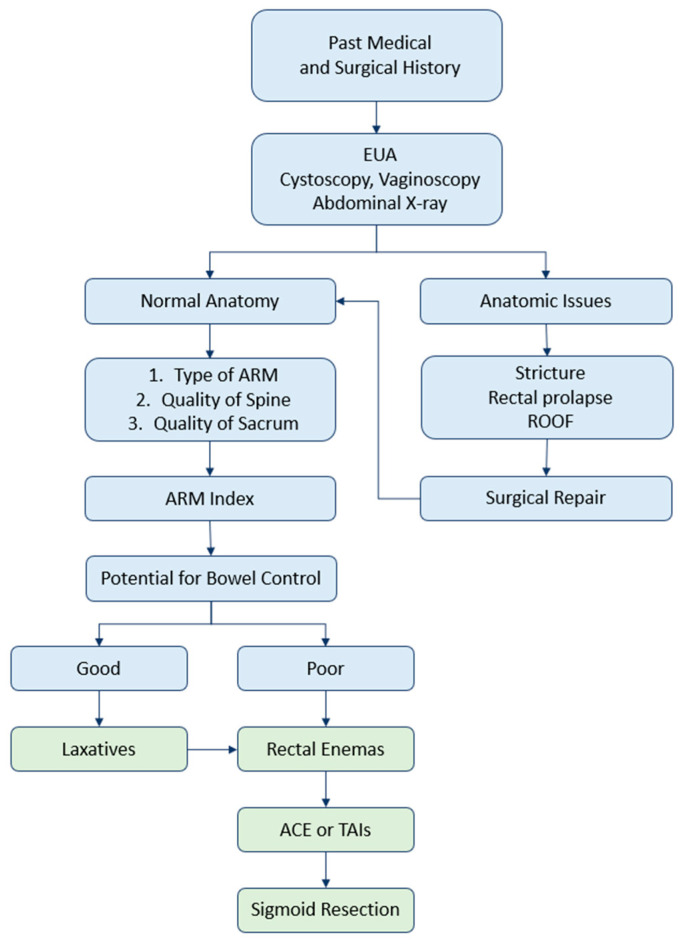
A stepwise protocol for the evaluation and management of patients with an anorectal malformation after the initial PSARP. ACE—antegrade continence enema; ARM—anorectal malformation; EUA—examination under anesthesia; ROOF—remnant of the original fistula; TAIs—transanal irrigations.

At a long-term follow-up after reconstruction, some ARM patients have stooling concerns and require bowel management [48]. In patients with a “low-” or “high-type” ARM, constipation was reported to be the most frequent complication after a PSARP (64.5% and 78.6%, respectively) [13]. A total of 67% of ARM patients who have visited multidisciplinary centers enroll in dedicated bowel management programs [49]. A stepwise protocol for the evaluation and management of patients with an ARM after the initial PSARP is demonstrated in Figure 3.

After anatomic variations have been evaluated and treated, an abdominal radiograph is an essential tool in the immediate and long-term management of constipation [13]. Patients must also be assessed for a megarectosigmoid that can cause long-standing constipation, recurrent impactions, and overflow incontinence [50]. The goal of therapy is to empty the colon daily (as confirmed by radiograph) without passage of stool between the regimen administration (laxatives, rectal enemas, transanal irrigations or antegrade flushes) and to have the child in regular underwear. The distribution of regimen used in ARM patients is similar in the recent studies: about 60% are managed mechanically with rectal or antegrade enemas, while approximately 35% are on laxatives [51,52]. Very rarely does a sigmoid resection need to be performed; with consistent laxative treatment, rectal enemas, transanal irrigations, or antegrade flushes, the colon function almost always improves.

### 5.1. Laxatives

If the underlying problem is constipation, and the patient has good potential for bowel control anatomically, stimulant laxatives should help push the stool through the colon, stimulating colonic propulsion waves [53]. Some patients may require larger doses than their counterparts due to a dilated rectosigmoid. Stimulant laxatives, such as Senna, are safe when used as a long-term medical treatment for constipation despite many myths to the contrary; doses are titrated during subsequent visits based on patient symptoms and abdominal radiograph findings [10]. The described complications include abdominal cramping, vomiting, or fecal incontinence, which are resolved after the change of the laxative, or a switch to rectal enemas [10]. More concerning is the perineal blistering mentioned by several authors [54,55,56,57,58,59,60,61]; however, it occurs on inconsistent or high-dose senna administration (60 mg/day), and it is associated with night-time accidents and prolonged stool-to-skin contact [10]. With a stepwise dosing of senna, the prevalence of perineal blistering was shown to be as low as 2.2% [10]. Given the need for formed stools to provide the appropriate sensation, and therefore, stay clean, stimulant laxatives that provoke a stool, complemented by a water-soluble fiber bulking to the stool, are the ideal combination. Stool softeners should be avoided in patients with an ARM [62], as loose stools are more difficult to detect given the impaired sensation.

### 5.2. Rectal Enemas

Rectal enemas should be started every 24 hours at the same time to allow for regular colonic emptying. As the stooling pattern improves, the frequency can be gradually decreased [63]. The solution should include saline, with a start volume of 400 mL. In children 3 years of age and older, saline can be substituted with tap water, as in younger patients, tap water can cause dehydration and hyponatremia [64]. If needed, stimulants (castile soap, glycerin, or bisacodyl) can be added. In the case of soiling between the enemas, the concentration of the stimulant should be decreased as overstimulation of the colon causes increased contractility, and therefore, frequency of bowel movements [64]. 

One of the difficulties patients with an ARM can face when performing a rectal enema is the inability to hold the retrograde flush due to maldeveloped anal sphincters, and thus, incomplete filling of the colon with the solution and decreased efficacy of the regimen. These children can be recommended transanal irrigations (TAIs) or antegrade flushes. The TAI system with a cone tip or a balloon helps the patients to hold the flush, while with antegrade enemas, the flush starts from the right colon and empties the bowel in an antegrade manner with no need to hold the solution for colonic emptying [65].

### 5.3. Transanal Irrigations

The transanal irrigation system (Peristeen^®^, Coloplast Denmark A/S, Humlebaek, Denmark) has gained popularity in patients with colorectal diseases due to increased independence when using this regimen [66], as well as the avoidance of surgical intervention in comparison to an antegrade continence enema procedure. The procedure takes 35 (12–60) minutes per day [66]. At a one-year follow-up with regularly administered transanal irrigations, 84% of compliant ARM patients are clean, while 16% reported 1–2 episodes of soiling per week [66]. 

TAI administration significantly improves the quality of life and constipation in patients with ARMs [67]; however, their administration can be challenging. More than half of ARM patients experience difficulties using TAIs or anorectal pain at the beginning of TAI administration [68]. However, these rates decrease significantly with the experience in TAI administration gained after 3 months (57% vs. 12%; 38% vs. 5%, respectively) [68]. The performance of at least one TAI after nurse supervision and initial training of the families to use the irrigation system at home increases the compliance, and therefore, continuity of TAIs [69]. For further information on transanal irrigations, see the related manuscript “State of the Art Bowel Management for Pediatric Colorectal Problems: Spinal Anomalies”.

### 5.4. Antegrade Continence Enemas

Antegrade continence enemas (ACEs) allow for administration of the flush from the right colon into the distal colon in an antegrade manner, which is a more physiologic in comparison to the retrograde administration of enemas/irrigations. Peeraully et al. reported 78% of children with an ARM achieving “success” on ACEs with or without minor leakage of stool the night after enema administration, and 13% reported improvement of their stooling pattern, with fecal continence not achieved yet [70]. The current updates on ACEs in bowel management are described in a related article “Pediatric Bowel Management Options and Organizational Aspects” [71]. 

### 5.5. Sigmoid Resection

Patients with refractory constipation can develop megarectosigmoid contributing to worsening constipation and overflow fecal incontinence [72]. Despite participation in a dedicated BMP, these patients might not improve on medical management only and can require a sigmoid resection [72,73]. Borg et al. described 26 patients managed for severe refractory constipation and a megarectosigmoid. Half of the patients were treated conservatively, while 12 patients underwent a sigmoid resection at a median age of 4.7 years (1.4–6.5) [74]. Reduction in the Grade 3 constipation rate was reported in both surgical and conservative groups based on the Krickenbeck classification at 5, 10, and 15 years of age (64–73%, 44–55%, and 18–25%, respectively) [21,74]. The sample size of the article did not allow for the definition of a significant correlation between the two treatment options; however, it leads to a conclusion that megarectosigmoid in patients after a PSARP could be managed conservatively without the risk of increased fecal incontinence and the need for a surgical procedure associated with a sigmoid resection. 

## 6. Reoperative (Redo) Procedures—Maximizing the Anatomy

### 6.1. Indications

After assessing a child’s anatomy, re-operative procedures with complete preoperative evaluation and long-term postoperative multidisciplinary care may be required [8,75]. In some patients, performing a redo procedure for correcting abnormalities/complications is the main factor needed to achieve fecal continence. A single-institution cohort study of success rate and quality of life after redo-PSARP included 153 patients who underwent a redo PSARP for an anoplasty mislocation, stricture, ROOF, or rectal prolapse (7%). One year following the redo procedure and correction of their anatomic problem, 49% of the patients with a good prognosis for bowel control had voluntary bowel movements but required the use of stimulant laxatives, 76% became completely continent of stool, and 51% of children had at least one episode of fecal incontinence per week despite being treated with enemas. Surprisingly, 20% of patients with expected poor continence potential (those children with an unfavorable anorectal anomaly, poor spinal, and/or sacral development) were rendered continent on stimulant laxatives alone and demonstrated the capacity for voluntary bowel movements. Overall, 77% of patients were continent in conjunction with a bowel management program and laxative use. The patients’ quality of life (76.7 vs. 83.8) and Baylor continence (29.2 vs. 17.7) scores also improved after redo surgery [8]. 

### 6.2. Simultaneous ACE Creation

In patients with poor potential for bowel control, a simultaneous ACE procedure can be performed at the time of a redo PSARP [8]. ACE creation should be considered in patients who will require long-term enema treatment or those who cannot tolerate the per rectal route [8]. The urologic plan should be revised preoperatively to assess the patient’s need for the creation of a simultaneous urinary catheterizable channel (Mitrofanoff or Monti) [76,77] and make an intraoperative decision on what type of ACE should be created [78,79,80,81,82]. For further information, see the related article “Pediatric Bowel Management Options and Organizational Aspects”.

## 7. Heineke–Mikulicz-like Anoplasty

Until recently, routine postoperative anal dilations have been recommended for all patients after a primary PSARP to prevent a postoperative stricture [83,84,85,86]. In 2021, Ahmad et al. reported no significant reduction in stricture formation in patients dilated after reconstruction [87]. Patients who develop a post-PSARP skin-level anal stricture can be managed with a Heineke–Mikulicz-like anoplasty (HMA). Halleran et al. described twenty-eight patients who underwent an HMA procedure at a mean age of 5.8 (0.5–24.4) years. The average preprocedural anal size was a Hegar 8 dilator, which was increased to a Hegar 16 after HMA. Modifying the HMA for such a situation performed for stricture development may offer an alternative to anal dilations and improve constipation symptoms [88,89].

## 8. Prognosis

A dedicated bowel management program was proven to significantly improve functional outcomes and quality of life (QoL) in children with colorectal diseases [90]. The focus of the current paragraph is the impact of a BMP on ARM patients. For information on other patient groups (Hirschsprung disease, functional constipation, and spinal anomalies), please refer to the related manuscripts.

Patients with an ARM who were enrolled in a bowel management program after a PSARP showed significant improvement in continence, self-confidence, and QoL [90]. At the end of a bowel management week, 88% of ARM patients adherent to treatment are clean on enemas, while 97% are successfully managed with laxatives [91]. The percentage of success slightly decreases over the first year (80% vs. 2% at six and twelve months, respectively) and remains constant after a one-year follow-up with a success rate of 71% at two years [91]. 

**Figure 4:**
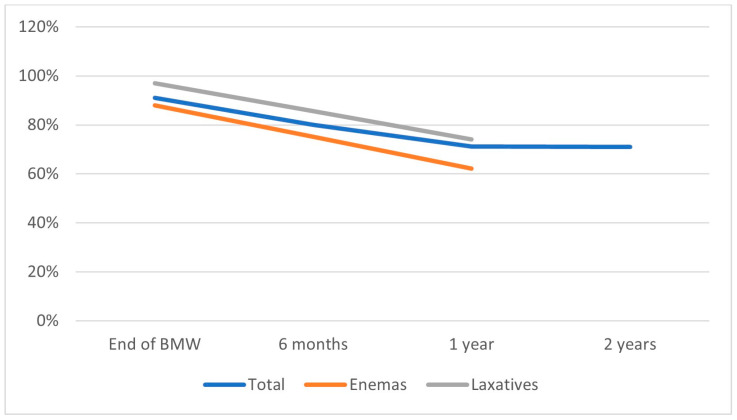
Continence rates in ARM patients involved in a dedicated bowel management program at the end of the bowel management week, at six months, one year, and two years based on the data reported by Wood et al. [52] and Kilpatrick et al. [91].

Wood et al. reported that 70% of 222 patients that were enrolled in a bowel management program for fecal incontinence were clean at a 1-year follow-up. Of these, 74% were successfully treated with enemas, and 62% of children on laxatives had voluntary bowel movements and were clean for stool. The study also demonstrated significant improvement in the Baylor, Vancouver, and total PedsQL scales from referral to one-year follow-up [52]. The summary of the BMP outcomes based on the literature is demonstrated in Figure 4.

Until recently, the heterogeneity in reporting the outcomes of bowel management and the variability of protocols for bowel management applied in different institutions did not allow the creation of a general idea of long-term outcomes in colorectal patients [92]. In 2018, The Pediatric Colorectal and Pelvic Learning Consortium (PCPLC) was established with a similar bowel management strategy, outcomes assessment, and follow-up applied in the participating institutions [93]. This novel structured collaborative approach and homogenous data reporting system will allow for further multi-institutional studies of long-term outcomes in patients with an ARM, Hirschsprung disease, functional constipation, and spinal anomalies.

## 9. Conclusions

After a primary PSARP, patients with an ARM can experience constipation and/or soiling and require bowel management. The evaluation includes examination under anesthesia and a contrast study to exclude anatomic causes of bowel dysfunction and screening for associated VACTERL malformations. Sacral X-rays and a spinal ultrasound or MRI are performed to define the potential for bowel control based on the calculated ARM index. The management options include laxatives, rectal enemas, transanal irrigations, and antegrade flushes. Stool softeners should be avoided due to maldeveloped anal sphincters, making it harder for these patients to detect soft stool. A redo PSARP might be needed to correct the anatomy and improve bowel function. Patients with mild stenosis can benefit from a Heineke–Mikulicz anoplasty instead of a more invasive redo PSARP.

## Figures and Tables

**Table 1:** Rome IV criteria for constipation in infants and children [15].

Rome IV Criteria for Constipation in Infants and Children *
<3 bowel movements per week
Straining during more than 25% of defecations
Lumpy or hard stools (Bristol Stool Form Scale 1–2) more than 25% of defecations
Sensation of incomplete evacuation more than 25% of defecations
Sensation of anorectal obstruction/blockage more than 25% of defecations
Manual maneuvers to facilitate more than 25% of defecations (e.g., digital evacuation, support of the pelvic floor)
Loose stools are rarely present without the use of laxatives
Insufficient criteria for irritable bowel syndrome

* Child must have ≥2 of the following criteria that are present for at least 3 months with symptom onset at least 6 months prior to diagnosis.

## Data Availability

No new data were created or analyzed in this study. Data sharing is not applicable to this article.
